# A ddRAD Based Linkage Map of the Cultivated Strawberry, *Fragaria xananassa*

**DOI:** 10.1371/journal.pone.0137746

**Published:** 2015-09-23

**Authors:** Jahn Davik, Daniel James Sargent, May Bente Brurberg, Sigbjørn Lien, Matthew Kent, Muath Alsheikh

**Affiliations:** 1 Norwegian Institute for Bioeconomy Research, Ås, Norway; 2 Fondazione Edmund Mach, Centre for Research and Innovation, San Michele all'Adige, Italy; 3 Center for Integrative Genetics, Department of Animal and Aquacultural Sciences, Norwegian University of Life Sciences, Ås, Norway; 4 Graminor Breeding Ltd., Ridabu, Norway; Department of Plant Sciences, Norwegian University of Life Sciences, Ås, Norway; Wuhan Botanical Garden of Chinese Academy of Sciences, CHINA

## Abstract

The cultivated strawberry (*Fragaria* ×*ananassa* Duch.) is an allo-octoploid considered difficult to disentangle genetically due to its four relatively similar sub-genomic chromosome sets. This has been alleviated by the recent release of the strawberry IStraw90 whole genome genotyping array. However, array resolution relies on the genotypes used in the array construction and may be of limited general use. SNP detection based on reduced genomic sequencing approaches has the potential of providing better coverage in cases where the studied genotypes are only distantly related from the SNP array’s construction foundation. Here we have used double digest restriction-associated DNA sequencing (ddRAD) to identify SNPs in a 145 seedling F_1_ hybrid population raised from the cross between the cultivars Sonata (♀) and Babette (♂). A linkage map containing 907 markers which spanned 1,581.5 cM across 31 linkage groups representing the 28 chromosomes of the species. Comparing the physical span of the SNP markers with the *F*. *vesca* genome sequence, the linkage groups resolved covered 79% of the estimated 830 Mb of the *F*. ×*ananassa* genome. Here, we have developed the first linkage map for *F*. ×*ananassa* using ddRAD and show that this technique and other related techniques are useful tools for linkage map development and downstream genetic studies in the octoploid strawberry.

## Introduction

*Fragaria* is an important soft fruit genus, primarily due to the cultivation of the genetically complex garden strawberry (*Fragaria* ×*ananassa* Duch; 2*n* = 8*x* = 56). In 2012, the world production of strawberries exceeded 5 million tons and the crop was valued in excess of US$10 billion [1]. In addition to its aesthetic qualities and nutritional value, strawberry is appreciated for its flavor, aroma, and content of ‘health-benefitting’ antioxidant compounds [2].

The cultivated strawberry is a genetically complex allo-octoploid (2*n* = 8*x* = 56). Very recent studies of the sub-genome structure of the species have determined that at least three diploid donors have contributed to the extant genome composition. The genome contains one sub-genome which displays similarity to *Fragaria iinumae*, two additional sub-genomes that are not clearly distinguishable, but that are clearly segregating disomically, and a single sub-genome with a strong similarity to *F*. *vesca* [3]. In the newly proposed model, the *F*. *iinumae* and undefined sub-genomes existed initially as a hexaploid, which subsequently hybridized at a much later stage with an *F*. *vesca*-like diploid to form the extant octoploid genome configuration [3]. The proposed sub-genome structure of *F*. *×ananassa* has thus been revised as A-A, b-b, X-X, X-X, where the A genome is *F*. *vesca*-like, the b genome is *F*. *iinumae*-like, and the two X genomes are of unknown origin but are delimited X-X, X-X to reinforce that they segregate independently in a disomic fashion, and may or may not be derived from the same ancestral progenitor.

The first sequence characterized linkage maps of the cultivated strawberry were constructed using microsatellites (simple sequence repeats; SSRs) [4–8]. However, the recent development of a high-throughput whole-genome genotyping array for *F*. *×ananassa* has provided researchers with a more powerful resource for the rapid development of dense linkage maps of the cultivated strawberry. Bassil et al [9] described the development of the SNP-array, IStraw90, and demonstrated its utility in the development of a linkage map of a progeny used in the array development SNP discovery process. The linkage map produced contained a total of 6,594 SNP markers distributed throughout 35 linkage group fragments that represented the 28 chromosomes of *F*. *×ananassa*. Whilst most of the chromosomes were well covered with markers, linkage groups 1D, 2C, 4C and 7C were significantly shorter than their homeologues on the linkage map. As an explanation, the authors reported that these sections largely corresponded to regions identified as homozygous on previous SSR maps developed for the mapping population [8].

The IStraw90 array should prove to be a powerful tool for genetics studies in the cultivated strawberry, however, the cost per sample of the array has implications for its applicability as a broad genotyping tool, both in very large experiments where the total cost of its implementation would be prohibitive, as well as in exploratory or pilot studies where initial proof of concept funding might be limited. In these scenarios, other high-throughput genotyping techniques may be more cost-effective for SNP genotyping and linkage map construction. Such approaches are based on genomic DNA enrichment using restriction digestion and adaptor ligation, followed by second-generation sequencing of multiplexed libraries. These techniques, along with others such as the direct re-sequencing of a sub-set of progeny individuals [10], allow the rapid generation of large numbers of single nucleotide polymorphisms (SNPs) for use in genetic analyses and genotyping. Genotyping-by-Sequencing (GBS), described by Elshire et al [11], has been used effectively to develop linkage maps of relatives of the cultivated strawberry including the diploid strawberry *F*. *vesca* [12], red raspberry [13], and apple [14], while RAD-tag sequencing, described by Baird et al [15], has been used to develop maps of barley [16] and aubergine [17] amongst others. These techniques have advantages over the use of array technology in that no *a priori* knowledge of the genomes of the organism under investigation is required, and are thus not dependent on previously identified SNPs being present in the genome of the study organism. They are also much cheaper per sample to assay than the use of arrays, but have the disadvantage of returning fragmented datasets containing high percentages of missing data. However, this might be resolved by imputation strategies to reduce the noise in the data used for map construction [13].

Tennessen et al [18] employed the targeted capture of DNA using RNA-derived baits that were subsequently sequenced using short-read sequencing technology to develop linkage maps for the two progenitor species of *F*. *×ananassa*. Whilst it has recently been reported that GBS libraries have been constructed for the cultivated strawberry [19], to date, no reports have emerged of GBS, RADseq, or any similar techniques having been used to develop linkage maps for *F*. *×ananassa*. In this investigation, we tested the efficacy of a modified RAD-tag protocol using double digestion with two restriction enzymes (ddRAD; [20]) for linkage map construction in the cultivated strawberry and present the first ddRAD based linkage map of cultivated strawberry, using an F_1_ population derived from the cross ‘Sonata’ × ‘Babette’ (S×B).

## Material and Methods

### Plant material and DNA extraction

An experimental population comprising 145 F_1_ hybrid seedlings was raised from the cross between the Dutch cultivar Sonata (♀) and the Norwegian cultivar Babette (♂). The F_1_ seed were germinated in mist chambers before being transplanted to flats and subsequently to larger pots. Young leaf tissue from one representative of each of the 145 F_1_ progeny plants and the two parents was flash frozen in liquid nitrogen and lyophilized for DNA extraction. DNA was extracted using DNeasy Plant Mini Kit (Qiagen) according to the manufacturer’s protocol. The resulting DNA was quantified using a Qubit Fluorometer (Invitrogen, USA) and the quality was assessed subjectively by agarose gel electrophoresis.

### ddRAD library construction and genotyping

Individual, high-molecular weight DNA samples, were prepared for sequencing according to the ddRAD protocol of Peterson et al [20]. Briefly, 500ng DNA was digested with *Eco*RI and *Msp*I (NEB, USA) before being purified using AMPure XP Beads (Beckman Coulter, USA). Paired combinations of double-stranded adapters were used to uniquely tag samples which, after cleaning to remove un-ligated adapter, were quantified and pooled in equimolar amounts; one pool included 96 samples, another included 49 while a third included just the parental genotypes. All three pools were subjected to size selection using a Pippin Prep (Sage Scientific, USA) and fragments were separated using a 2% agarose cartridge to capture a narrow distribution around 400bp. Sequencing was performed using an Illumina MiSeq (Illumina, USA) and V2 sequencing kit chemistry (2×251 nt).

SNPs were detected using Stacks (v1.18) software [21]. Briefly, raw forward reads were normalized to a common length of 240 nt before being aligned with each other to form stacks; minimum stack depth 5 (-m), minimum distance allowed between stacks 4 (-M), all other parameters used default values. Data was exported from Stacks in JoinMap format.

### Linkage map construction

Data produced by Stacks were coded as an F_1_ segregating population using the genotypes of the parental lines to assign segregation. Data were filtered for all markers containing more than 50% missing values and a chi-squared analysis was performed to determine segregation distortion at the 5% level of significance (Chi-squared = 3.841 (1 d.f.), 5.991 (2 d.f.) or 7.815 (3 d.f.)). Initially, only robust markers for which no significant segregation distortion was observed at the 5% level were used for linkage mapping using JoinMap 4.1 (Kyazma, NL). An initial linkage map was constructed using the Maximum Likelihood mapping function and assessed for spurious linkages or inflated genetic distances, with individual genotypes being converted to missing values where necessary or loci being removed completely where they caused conflicts in the data. Following scrutiny of the Maximum Likelihood data, linkage mapping was performed with the initial marker set using regression mapping and v1.0 linkage groups were produced. Additional data were then added to the dataset for markers exhibiting significant segregation distortion. Marker placement was determined using regression mapping with a minimum logarithm of odds (LOD) score threshold of 3.0, a recombination fraction threshold of 0.35, ripple value of 1.0, jump threshold of 3.0 and a triplet threshold of 5.0, and mapping distances were calculated using the Kosambi mapping function to produce a v2.0 linkage map. Data from the v2.0 map were compared to the v1.0 linkage maps, and any markers causing shifts in the placement of the initial robust markers were removed from the linkage groups, following which, marker ordering was recalculated to produce the linkage map presented. Due to the level of missing values in the dataset, marker bins were calculated from map positions with no decimal places. The sequence tags associated with each SNP were used as queries for a BLAST analysis against the v2.0 (*Fvb*) genome sequence of *F*. *vesca* ‘Hawaii 4’ [22,23]. Linkage groups were identified and named according to the pseudo-chromosomes to which the mapped SNP sequence tags were associated and homeologous groups were arbitrarily assigned the suffixes A, B, C and D. The linkage maps presented were plotted using MapChart 2.1 [24]. Physical positions of the SNP sequence tags were used to plot MareyMaps of genetic vs. physical position of all mapped genetic markers.

## Results

### Data analysis and linkage map construction

A total of 66,318 stacks were detected from analysis of the forward reads, 20% of these (13,302) contained one or more SNPs. To maximize data completeness and minimize false positive SNPs, stacks were only included if they were detected in both parents and ≥ 100 offspring (≥ 69%) and had ≤ 5 SNPs within the 240 nt sequence. This resulted in a list of 1,098 SNP-containing stacks that were used for mapping.

**Fig 1:**
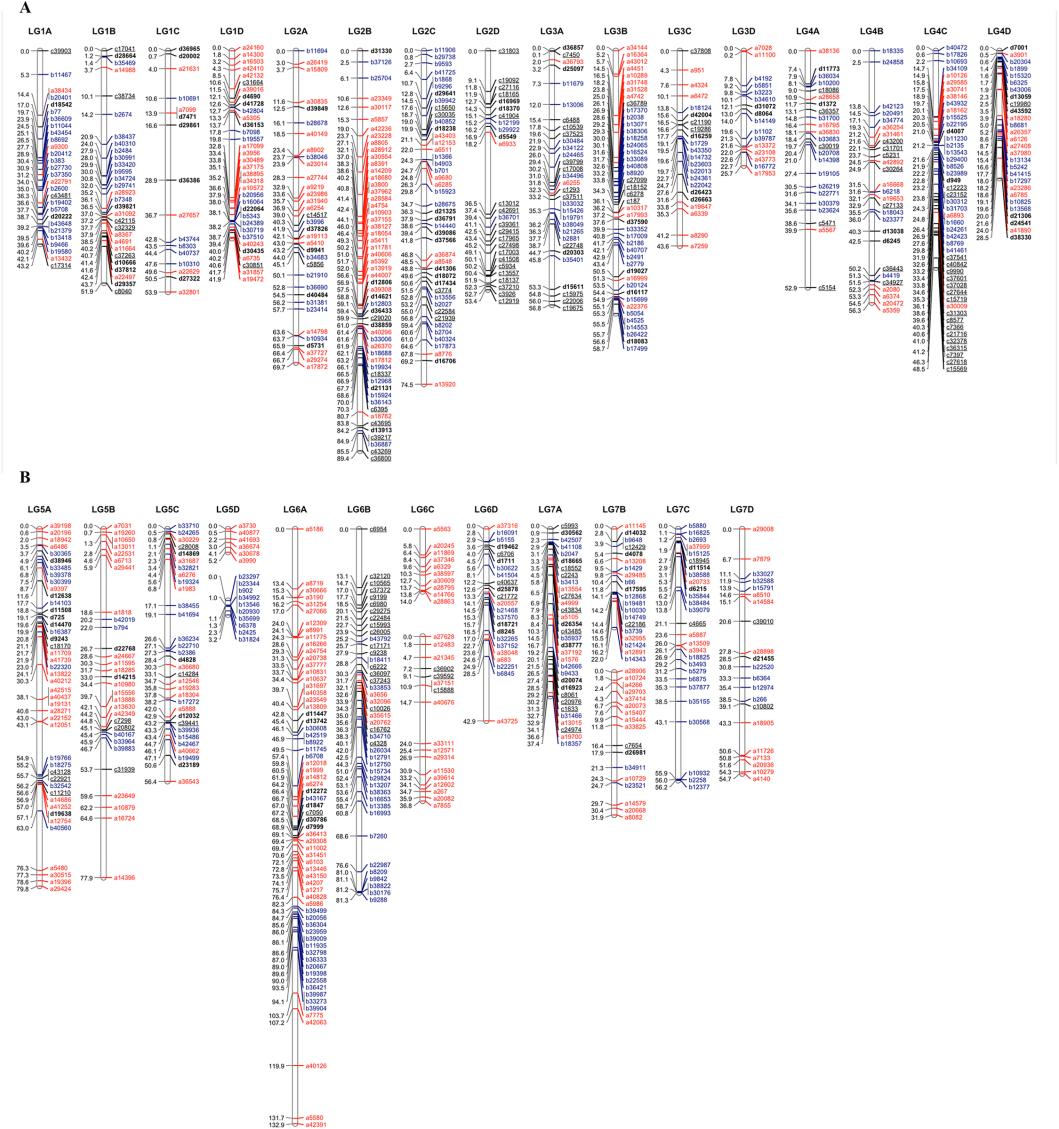
A SNP-based linkage map of a *F*. *×ananassa* mapping population derived from the cross ‘Sonata’ *×* ‘Babette’. Map distances are given in centi-Morgans (cM), marker colours indicate: Red–markers segregating in the ‘Sonata’ genetic background only; Blue–markers segregating in the ‘Babette’ genetic background only; Black–markers segregating in both genetic backgrounds (1:1:1:1 and 1:2:1 segregations are indicated with bold and underscore respectively).

Of the 1,098 SNPs, 59 did not segregate within the seedlings (they were called as SNPs due to the presence of a false homozygote call for a small number (≤ 3 of seedlings), whilst a further 306 exhibited significant segregation distortion. The remaining 733 were used to construct the initial v1.0 linkage maps. Following scrutiny of the resultant linkage groups, the markers exhibiting significant segregation distortion were added to the dataset and 1,039 markers were analyzed for linkage and marker ordering. The final linkage map produced contained a total of 902 sequence characterized SNP markers in 650 mapping bins spanning 1,581.5 cM across 31 linkage group fragments that corresponded to the full complement of 28 chromosomes of the *F*. *×ananassa* genome (Fig 1). The map resolution corresponded to one marker every 1.68 cM and on bin every 2.34 cM. Each chromosome was represented by a single linkage group except for three; LG5D, LG6C and LG7B, which were represented by two linkage group fragments each. The longest linkage group was LG6A (132.9 cM), whilst the shortest was LG5D2 (3.2 cM). Overall, a slightly higher proportion of markers were heterozygous in ‘Sonata’ than in ‘Babette’ (67.7% vs. 61.4% respectively). Moreover, the distribution of heterozygous markers was not uniform across the genomes. The proportion of heterozygous markers on individual linkage groups ranged from 32.1% and 29.4% to 94% and 96.2% in ‘Sonata’ and ‘Babette’ respectively.

**Fig 2:**
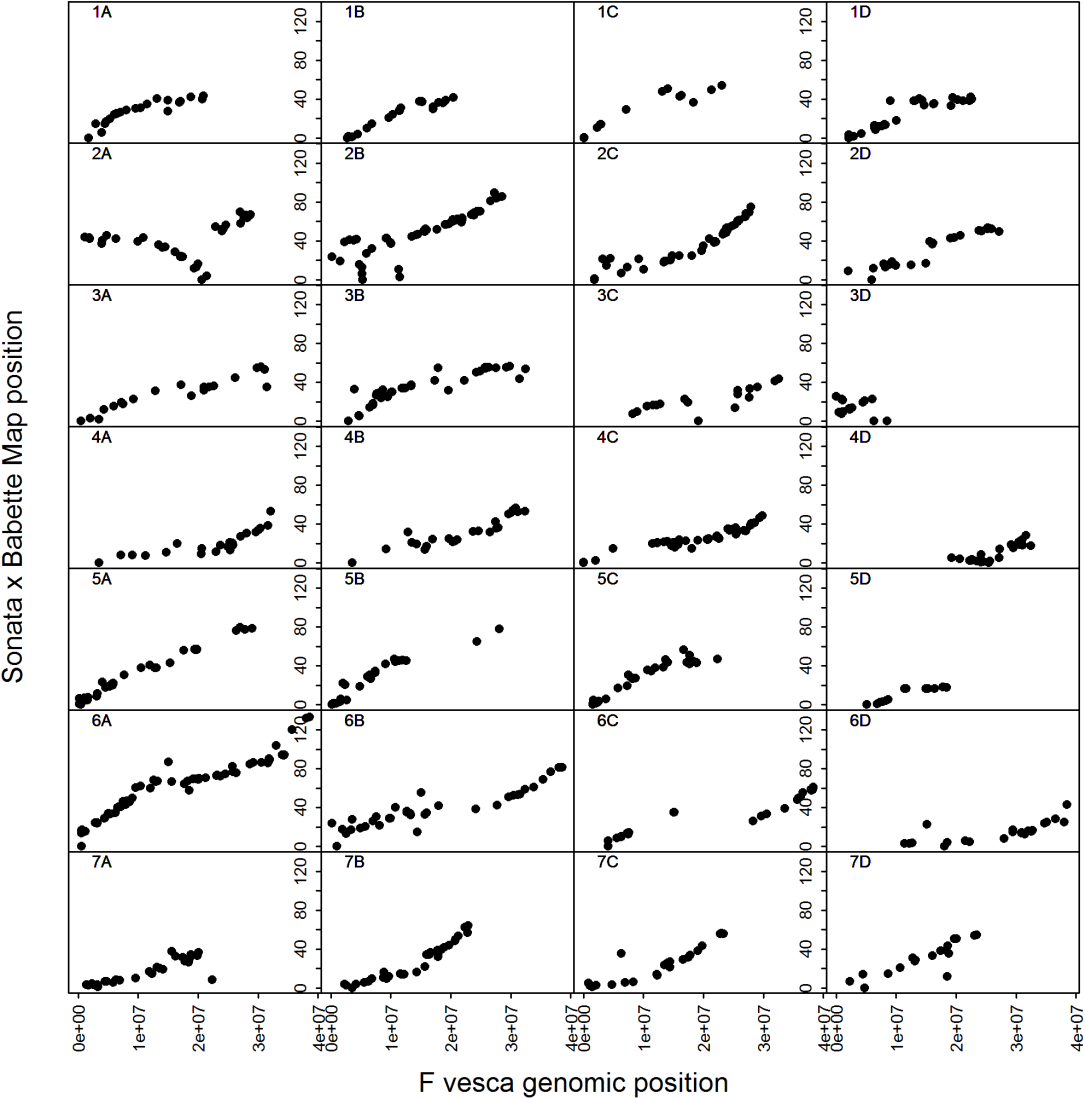
Marey Map plots of SNPs mapped to positions on the 28 *F*. *×ananassa* chromosomes vs. their physical positions on the v2.0 *F*. *vesca* (*Fvb*) pseudomolecules. Linkage sub-group fragments for groups LG5D, LG6C and LG7B have been joined with an artificial gap of 10 cM between fragments to facilitate data visualization.

Through comparison to the physical span of the SNP markers on the *Fvb* genome sequence, the linkage groups resolved covered 656.2 Mb (79%) of the estimated 830 Mb of the *F*. *×ananassa* genome size. Table 1 lists the lengths of the 31 linkage group fragments that comprise the S×B linkage map, along with the number of markers each contains, and the physical span of each group. Visualization of the genetic distances of the mapped SNPs vs. their physical positions on the *Fvb* genome sequence on each linkage group (Fig 2) revealed a generally a high degree of collinearity, however, putative large-scale inversions/rearrangements were observed on the distal section of LG2A and 2B.

**Table 1:** Summary statistics for the 31 linkage group fragments that comprise the S×B linkage map, including the total number of markers mapped per linkage group, the numbers and proportions of the different segregation classes, the proportion of markers heterozygous in each parental genome, linkage group lengths and the physical distances associated with each group on the v2.0 *F*. *vesca* genome sequence.

LG	LG length (cM)	Total no markers	Total no bins	AA×AB	AB×AA	AB×AB	AB×AC	Proportion of markers in Sonata	Proportion of markers in Babette	LG physical start (bp)*	LG physical end (bp)*	Total physical span (bp)*
1A	43.2	28	21	4	19	3	2	32.1	85.7	1796261	20896488	19100227
1B	51.871	29	21	7	11	6	5	62.1	75.9	2782522	20477424	17694902
1C	53.861	16	14	5	5	0	6	68.8	68.8	81441	23060693	22979252
1D	41.855	34	20	18	9	2	5	73.5	47.1	2096561	22681981	20585420
2A	69.718	34	29	17	10	2	5	70.6	50.0	1171481	28736730	27565249
2B	89.447	54	38	30	10	7	7	81.8	45.5	259771	28580973	28321202
2C	74.531	44	36	9	20	5	10	54.5	79.5	1847152	27959937	26112785
2D	53.423	26	18	1	3	19	3	88.5	96.2	2085375	27233633	25148258
3A	56.797	31	26	2	13	12	4	58.1	93.5	519132	31515269	30996137
3B	58.687	44	28	12	23	5	4	47.7	72.7	2994561	32529792	29535231
3C	43.559	22	18	7	8	3	4	63.6	68.2	8200628	32611773	24411145
3D	25.732	16	12	6	8	0	2	50.0	62.5	52228	8533244	8481016
4A	52.876	23	20	5	11	5	2	52.2	78.3	3456977	32172047	28715070
4B	56.335	27	20	9	9	7	2	66.7	66.7	3608485	32405198	28796713
4C	48.47	47	27	7	20	18	2	57.4	85.1	28438154	29816896	1378742
4D	28.494	28	15	9	12	1	6	57.1	67.9	19244446	32484863	13240417
5A	79.763	44	30	22	11	4	7	75.0	50.0	198137	28991129	28792992
5B	77.854	30	25	20	5	3	2	83.3	33.3	178230	28161007	27982777
5C	56.409	32	23	11	14	3	4	56.3	65.6	1540359	22337882	20797523
5D1	5.196	6	6	6	0	0	0	100	0	5146419	8718906	3572487
5D2	3.164	10	4	0	10	0	0	0	100	11458264	18444476	6986212
6A	132.871	67	43	39	21	1	6	68.7	41.8	561802	38607442	38045640
6B	81.304	43	34	4	21	18	0	51.2	90.7	240060	38607442	38367382
6C1	14.047	10	6	10	0	0	0	0	100	4133962	7585971	3452009
6C2	36.837	17	16	14	0	3	0	35.3	100	15102989	38305762	23202773
6D	42.934	23	16	5	10	3	5	56.5	78.3	11457218	38499401	27042183
7A	37.416	32	23	7	9	10	6	71.9	78.1	1413873	22338810	20924937
7B1	22.034	20	11	5	10	2	3	50	75	2344369	15726500	13382131
7B2	31.875	17	14	13	2	1	1	94.1	29.4	16028826	22918771	6889945
7C	56.189	27	19	5	18	2	2	33.3	81.5	811074	23361834	22550760
7D	54.719	21	17	11	7	2	1	66.7	47.6	2344358	23486539	21142181
Total	1581.468	902	650	320	329	147	106	61.4	67.7	n/a	n/a	656193698

^*^LG physical start: Position of the first mapped marker on the *Fvb* genome sequence. LG physical end: Position of the last mapped marker on the *Fvb* sequence. Total physical span: The distance between these markers.

## Discussion

The first linkage maps of *F*. *×ananassa* were developed using arbitrarily-primed markers such as amplified length-fragment polymorphisms [25]. Whilst these markers facilitated the development of linkage maps containing many markers spanning the majority of the *F*. *×ananassa* genome they were not sequence-characterized and thus not reliably transferrable between investigations. Second-generation linkage maps for the species incorporated SSR markers and other arbitrary marker types [26,27], or were exclusively composed of SSRs [4–6,8] which proved to be highly transferrable, even between linkage maps of different *Fragaria* species [28–30]. However, the relatively high development and screening costs of SSRs resulted in only a few studies producing saturated linkage maps for *Fragaria* species [6].

The availability of a reference genome sequence for *F*. *vesca* [22] and advances in re-sequencing technologies permitted the development of a high throughput whole genome genotyping array for *F*. *×ananassa* [9], which in turn led to the construction of ‘next-generation’ SNP-based linkage maps for the species with a minimum of experimental research effort [3]. Whilst the cost per SNP on such maps is low, the cost per sample is relatively high, making genotyping of large populations relatively expensive. Sequencing of reduced representation genomic libraries using short-read sequencing technology represents a compromise between sample screening cost-effectiveness and robust, abundant sequence-characterized marker genotyping.

Previously, we used GBS to develop a linkage map of a diploid *F*. *vesca* progeny for the purposes of studying disease resistance [12], and the technique has also been used to develop linkage maps of another close diploid relative of *F*. *×ananassa*, red raspberry [13]. Tennensen et al [18] used target-capture sequencing to develop linkage maps for octoploid *Fragaria* species, however, this is the first time, to our knowledge that a linkage map has been produced for one of the complex allo-octoploid *Fragaria* species using restriction-based genome enrichment techniques. We demonstrate here that robust linkage map development in the cultivated strawberry is achievable using the ddRAD genotyping approach first reported by Peterson et al [20]. Using two restriction enzymes and a strict fragment size limitation in the ddRAD method reduced the proportion of the genome sampled and led to a higher sequencing coverage. In theory, this reduces the number of false homozygote calls and missing values in the dataset produced. The approach led to a smaller number of identified segregating SNPs than in studies of other Rosaceous species [13], but with a higher proportion of those identified SNPs mapping to one of the linkage groups defined.

The S×B linkage map has a total genetic distance over the 31 linkage groups of 1,581.5 cM, which is comparable to other maps produced using regression mapping. The SNP-based ‘Darselect’ × ‘Monterrey’ F_1_ linkage map of Sargent et al [3] was 1,820 cM, and the SSR maps of Zorrilla-Fontanesi et al [27] 1,400.1 cM, and van Dijk [8] was 1846 cM. All these were smaller than the SSR map of Sargent et al [4] (2140.3 cM). The SNPs mapped in the S×B population displayed a high degree of collinearity with the v2.0 *F*. *vesca* (*Fvb*) pseudomolecules indicating that the map positions of the ddRAD markers was reliable. This, along with the total length of the linkage map, and the estimated proportion of the *F*. ×*ananassa* genome it covered (79%) indicates that the map represents the majority of the ‘Sonata’ and ‘Babette’ genomes, and will provide a useful resource for future studies of segregating traits of interest in the progeny. As in other *F*. ×*ananassa* linkage maps, the proportion of markers heterozygous in each of the parental genotypes varied between linkage groups, but no clear patterns were observed that would permit us to speculate on the effects of breeding and selection as in previous studies [5,8].

## Concluding Remarks

We have developed the first linkage map for *F*. *×ananassa* using ddRAD, a technique exploiting the power of short read sequencing technology and reduced representation genome coverage to call sequence variation in the progeny of a segregating mapping population. Whilst the number of markers we were able to score using this approach was less than when using the IStraw90 whole genome genotyping array, the map produced covered the genomes of the two parental cultivars adequately, spanning some 79% of the total estimated genome size, and placement of markers was robust and reliable, evidenced through a good correlation between the genetic positions of the markers mapped and their physical positions on the *Fvb* genome sequence. Our investigation provides clear evidence that ddRAD, and by extension, other related techniques such as RADseq and GBS, are useful tools for linkage map development in cultivated strawberry, and permit the rapid development of good quality linkage maps for downstream genetic studies of segregating traits.

## Supporting Information

S1 Table Presents tag number, segregation type, diploid chromosome homeolog, linkage group, diploid chromosome position, SNP position(s), and tag sequences. (XLSX)
